# Resection of Humerus

**Published:** 1896-04

**Authors:** W. W. Pugh

**Affiliations:** Hearne, Texas


For the Texas Medical Journal.

### RESECTION OF HUMERUS.

BY W. W. PUGH, M. D., HEARNE, TEX.

WHEN I consider the many "fads" coming before the surgeons in the last few years, I am not surprised that a great many are not accepted, and put in practice.

### SOME LATE PRINCIPLES ON BONE SURGERY.

I have reference to the early diagnosis and immediate application of surgical means to prevent the destruction of bone tissue. One case in particular, which has been of much interest to myself and others, to whom I have related the case.

Mr. McWilliams' little daughter, Ema, living near Hearne, Texas, three years ago, last May, had her os humerus broken five inches below the head of humerus, and another fracture three inches lower, or middle, of humerus, making a compound fracture. The bone was set by a physician, but union failed to take place, and the bone lay there in the arm, between the other portions of the humerus, as a foreign substance. The results were suppuration; caries, or death of flesh and bone, took place. I was sent for, (I was then living at Kingston, Texas,) last May to see the case. I found the little girl very much depleted, and suffering with blood poisoning, from absorbed septic matter, caused from decomposed flesh and bone. I at once advised the immediate removal of the loose bone, or "resection operation. The father hesitated, but finally consented to let me do the work; and with the assistance of my brother, Dr. T. J. Pugh, I at once proceeded. First, (seeing she had been poorly nourished, and also much emaciated, pulse 180, rapid breathing,) I gave her a large dose of quinine sulp. and strychnine sulp. hypodermically, then gave chloroform, just enough to destroy nerve sensibility. I then cut down directly longitudinaly with bone, and found the bone completely detached from the other two heads of the fractured bone. I removed the section of bone with fauceps, cleansed the wound thoroughly with antiseptic warm-water, examined the other two heads of bone, leaving a space of 2½ or 3 inches, without any bone at all, and found the two heads completely smooth, and they looked as there would be no chance for bony substance to be thrown out to cause re-union later on. Nevertheless, I was hopeful of this, knowing the wonderful effort nature always makes in repairing itself. I dressed the wound aseptically, and returned to Hearne. Next day my brother and I went to see the patient; found her resting quietly, and she stated she felt better than she had for three years. Pulse 85, and respiration good, and she was taking some nourishments. I advised the hypophosphates, as a tonic and bone builder. Came back to Hearne. The next day I returned to my home at Kingston, leaving the case with my brother. Immediately after locating at Hearne, I sent for the girl, Ema McWilliams, in order that I could learn how she was getting along.

She is now about thirteen years old, and she is a new girl, compared to what she was ten months ago. I find, from careful examination, that there has complete union taken place, but a slight ankalosed joint at the elbow, which existed when I first saw the case. Later on I shall endeavor to break up this stiffness, if the parents will allow me to do so.

Is there another case on record of union having taken place after a lapse of three years? I think it quite a remarkable case, although the girl is young. I have observed, after removing a portion of the larger part of the longer bones, leaving the periostium, the bone was reproduced enough to fill the cavity, and make a good, strong, useful limb, where there is a great destruction of bone, especially of foot, finger, hand and arm, by the proper use of warm water, to prevent death, and the application of aseptic dressing, taking every precaution not to obstruct even the circulation of the skin, applying oiled silk and iodoform gauze, and the free use of surgical cotton, which concentrates hemorrhage and union by first intention, also retaining the bones in proper position, preserving the temperature and circulation, while aided by positives, etc. I have also seen the bones entirely restored to the former usefulness. Observing these facts, I am of the opinion that as soon as you decide you will have death of a bone, you should in sist upon removing all of that portion then apply hot water, and dress as above suggested, and not wait for its pernicious infection to extend to the adjacent joints, or destroy any unnecessary bone or tissue. I have seen amputation that, according to my opinion, might have been prevented if this had been the guide post. I never put in any tubes at the lower or upper part of the wound to irrigate. I allow nature to do its own good work. I have not lost a case of surgery since I commenced practice, in 1885. During this time I have performed several operations in different parts of the State.

